# Anticancer Potential of *Cymbopogon citratus* L. Essential Oil: In Vitro and In Silico Insights into Mitochondrial Dysfunction and Cytotoxicity in Cancer Cells

**DOI:** 10.3390/plants14091341

**Published:** 2025-04-29

**Authors:** Tamara Maksimović, Daliana Minda, Codruța Șoica, Alexandra Mioc, Marius Mioc, Daiana Colibășanu, Alexandra Teodora Lukinich-Gruia, Maria-Alexandra Pricop, Calin Jianu, Armand Gogulescu

**Affiliations:** 1 Department of Pharmacology-Pharmacotherapy, Faculty of Pharmacy, “Victor Babes” University of Medicine and Pharmacy, Eftimie Murgu Square, No. 2, 300041 Timișoara, Romania; tamara.maksimovic@umft.ro (T.M.); codrutasoica@umft.ro (C.Ș.); 2 Research Center for Experimental Pharmacology and Drug Design (X-Pharm Design), “Victor Babes” University of Medicine and Pharmacy, Eftimie Murgu Square, No. 2, 300041 Timișoara, Romania; marius.mioc@umft.ro (M.M.); daiana.handa@umft.ro (D.C.); 3 Department Pharmacognosy-Phytotherapy, Faculty of Pharmacy, “Victor Babes” University of Medicine and Pharmacy, 2 Eftimie Murgu, 300041 Timisoara, Romania; daliana.minda@umft.ro; 4 Department of Pharmaceutical Chemistry, Faculty of Pharmacy, “Victor Babes” University of Medicine and Pharmacy, Eftimie Murgu Square, No. 2, 300041 Timișoara, Romania; 5 OncoGen Centre, Clinical County Hospital “Pius Branzeu”, Blvd. Liviu Rebreanu 156, 300723 Timisoara, Romania; alexandra.gruia@hosptm.ro (A.T.L.-G.); alexandra.pricop@oncogen.ro (M.-A.P.); 6 Department of Applied Chemistry and Environmental Engineering and Inorganic Compounds, Faculty of industrial Chemistry, Biotechnology and Environmental Engineering, Polytechnic University of Timisoara, Vasile Pârvan 6, 300223 Timisoara, Romania; 7 Faculty of Food Engineering, Banat’s University of Agricultural Sciences and Veterinary Medicine “King Michael I of Romania” from Timisoara, Calea Aradului 119, 300629 Timișoara, Romania; calin.jianu@gmail.com; 8 Department XVI: Balneology, Medical Rehabilitation and Rheumatology, “Victor Babes” University of Medicine and Pharmacy, 2 Eftimie Murgu, 300041 Timisoara, Romania; gogulescu.armand@umft.ro

**Keywords:** lemongrass (*Cymbopogon citratus* L.) essential oil, anticancer activity, natural compounds, molecular docking

## Abstract

This study aims to assess the potential anticancer activity of lemongrass essential oil (LEO) using in vitro and in silico methods. The steam hydrodistillation of the aerial parts yielded 3.2% (wt) LEO. The GC-MS analysis of the LEO revealed the presence of α-citral (37.44%), β-citral (36.06%), linalool acetate (9.82%), and d-limonene (7.05%) as major components, accompanied by several other minor compounds. The antioxidant activity, assessed using the DPPH assay, revealed that LEO exhibits an IC_50_ value of 92.30 μg/mL. The cytotoxic effect of LEO, as well as LEO solubilized with Tween-20 (LEO-Tw) and PEG-400 (LEO-PEG), against a series of cancer cell lines (A375, RPMI-7951, MCF-7, and HT-29) was assessed using the Alamar Blue assay; the results revealed a high cytotoxic effect against all cell lines used in this study. Moreover, neither one of the tested concentrations of LEO, LEO-PG, or LEO-TW significantly affected the viability of healthy HaCaT cells, thus showing promising selectivity characteristics. Furthermore, LEO, LEO-PG, and LEO-TW increased ROS production and decreased the mitochondrial membrane potential (MMP) in all cancer cell lines. Moreover, LEO treatment decreased all mitochondrial respiratory rates, thus suggesting its ability to induce impairment of mitochondrial function. Molecular docking studies revealed that LEO anticancer activity, among other mechanisms, could be attributed to PDK1 and PI3Kα, where the major contributors are among the minor components of the essential oil. The highest active theoretical inhibitor against both proteins was β-caryophyllene oxide.

## 1. Introduction

Anticancer treatments have advanced significantly in recent decades, with novel emerging therapies triggered by the inherent challenges to conventional agents such as multidrug cell resistance and severe side effects [1]. Although providing superior clinical outcomes, such new strategies come with high costs and challenges of their own (i.e., ability to evade the immune response, development of efficient and biocompatible delivery systems, etc.) [2]. Given that cancer retains high incidence and mortality worldwide, making it a leading cause of death [3], there is continuous pressure to develop new agents able to provide more effective and selective alternatives for cancer treatment. Plants have provided numerous molecular scaffolds with anticancer efficacy but also various compounds valuable as adjuvants during chemotherapy, able to synergize with antitumor drugs in terms of acting as drug sensitizers, reducing resistance to drugs or alleviating drug side effects [4].

Essential oils are mixtures of volatile secondary metabolites of plant origin that have developed as part of the plant’s chemical defense system against external threats such as herbivores, insects, or microorganisms, and they exhibit intrinsic pharmacological properties. They have been used extensively for the prevention and treatment of various diseases, including cancer [5]. Essential oils display a complex chemical composition containing up to 300 different molecules, among which phenols, alcohols, and aldehydes were found to induce the strongest antitumor properties by means of various mechanisms including cancer prevention, direct effects against cancer cells, and the tumor microenvironment [6].

Lemongrass essential oil (LEO) from *Cymbopogon flexuosus* was tested as an anticancer agent against colon, lung, cervix, oral, prostate, and leukemia cancer cells and showed strong cytotoxic effects through dose-dependent apoptosis; the in vitro effects correlated with the in vivo dose-dependent inhibition of tumor growth in the animal models of solid tumors [7]. LEO, as well as its main constituents citral and geraniol, were identified as effective antiproliferative agents in MCF7 breast cancer cells through the inhibition of HSP90 chaperone protein [8]; additionally, citral exhibits selective cytotoxic activity against breast cancer cells [9]. Citral induces multiple anticancer molecular mechanisms that include the accumulation of ROS in cancer cells with subsequent DNA damages and the inhibition of tubulin polymerization and aldehyde dehydrogenase isoform ALDH1A3, which favors cancer proliferation and chemoresistance [10]; moreover, other minor components in lemongrass essential oil induce cytotoxic effects through various mechanisms. However, despite the numerous papers reporting the direct correlation between the anticancer activity of LEO and its citral content, one study published in 2020 by Viktorová et al. revealed that neither the anticancer nor the antimicrobial activity of LEO are caused by the presence of citral; the authors concluded that the natural mixture of compounds identified in LEO presumably induce synergistic effects, and their use as a whole is more beneficial that the use of pure citral [11].

The current study aims to assess the potential of LEO, extracted from *Cymbopogon citratus* L., to act as an anticancer agent against a series of cancer cell lines (A375, RPMI-7951, MCF-7, and HT-29); the comparative assessment with pure citral was conducted as well. Additionally, the underlying molecular mechanisms were investigated by in silico methods.

## 2. Results

### 2.1. Chemical Composition of LEO

The steam hydrodistillation of the aerial parts of *Cymbopogon citratus* L. yielded 3.2% (wt) of a pale yellow to light greenish-yellow oil with a characteristic citrus-like lemony odor. The GC-MS analysis of the LEO revealed the presence of α-citral (37.44%), β-citral (36.06%), linalyl acetate (9.82%), d-limonene (7.05%), and β-caryophyllene (6.73%) as major components, accompanied by several other minor compounds, as shown in Table 1.

### 2.2. Determination of the Antioxidant Activity of LEO

LEO antioxidant scavenging activity was determined by employing 2,2-diphenyl-1-picrylhydrazyl (DPPH) and [2,2′-azino-bis(3-ethylbenzthiazoline-6-sulfonic acid)] (ABTS) radical scavenging assays. Pure ascorbic acid (AA) and butylated hydroxyanisole (BHA) were used as positive controls in the DPPH assay. For the ABTS assay, 1 mg/mL solutions of AA and BHA were used as standard references to determine percentage inhibition. The results are presented in Table 2.

### 2.3. LEO Effect on Cell Viability

**Figure 1:**
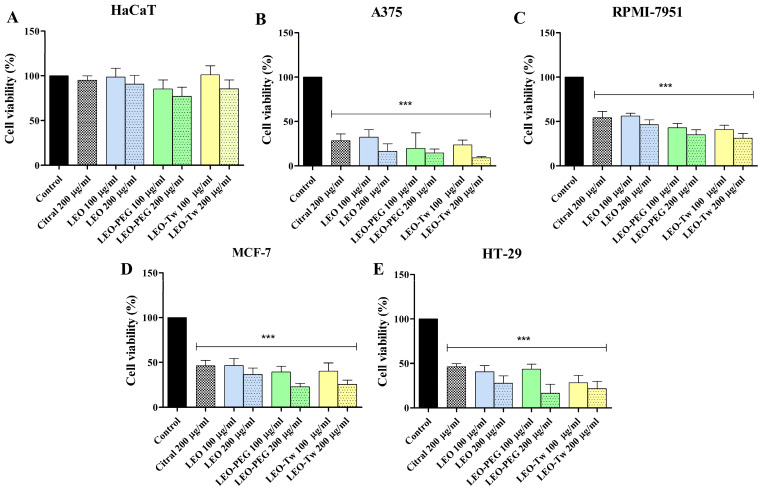
Cellular viability of HaCaT (**A**), A375 (**B**), RPMI-7951 (**C**), MCF-7 (**D**), and HT-29 (**E**) cells after 48 h stimulation with 100 µg/mL and 200 µg/mL simple LEO water dispersion, LEO-PEG, and LEO-Tw. The results are defined as cell viability percentage compared to control (100%) and expressed as mean values ± SD of three individual experiments (*** *p* < 0.001).

LEO presented diverse effects depending on the cell line and tested concentration. The HaCaT cells did not show a significant decrease in viability after LEO, LEO-PEG, and LEO-Tw treatments, even at the highest tested concentrations (200 µg/mL) (Figure 1A). The viability of all the cell lines treated for 48 h with PEG-400 and Tween 20 alone did not significantly differ from the control. Meanwhile, cellular viability was significantly affected in the cancer cell lines. A dose-dependent drop in viability was observed in the case of A375 cells, in which stimulation with 100 µg/mL and 200 µg/mL LEO resulted in 32.3 ± 6.1% and 16.5 ± 5.9% cell viability vs. control (100%); treatment with 100 µg/mL and 200 µg/mL LEO-PEG decreased the viability to 19.7 ± 12.3% and 14.6 ± 3.1%; and treatment with 100 µg/mL and 200 µg/mL LEO-Tw reduced the viability to 23.7 ± 3.9% and 9.2 ± 0.8% vs. 200 µg/mL citral (28.43 ± 6.65%) and vs. control (100%) (Figure 1B). Compared to A375, in the other melanoma cell line, RPMI-7951, citral, LEO, and LEO formulations decreased viability vs. control; however, it was to a lesser degree than in A375 as follows: 54.21 ± 7.13% (citral), 56.22 ± 3.11% (LEO 100 μg/mL), 46.42 ± 5.05% (LEO 200 μg/mL), 42.91 ± 5.51% (LEO-PEG 100 μg/mL), 35.13 ± 5.41% (LEO-PEG 200 μg/mL), 40.88 ± 4.90% (LEO-Tw 100 μg/mL), and 31.17 ± 5.38% (LEO-Tw 200 μg/mL) (Figure 1C). Similarly, HT-29 colorectal cancer cells were affected in a dose-dependent manner: 40.6 ± 5.1% and 27.8 ± 6.4% of the cells remained viable after 100 µg/mL and 200 µg/mL LEO treatment, respectively; the viability decreased to 43.7 ± 3.9% and 16.4 ± 7.1% after 100 µg/mL and 200 µg/mL LEO-PEG treatment and to 28.2 ± 6.0% and 21.6 ± 6.4% after 100 µg/mL and 200 µg/mL LEO-Tw treatment. Citral decreased HT-29 cell viability to 46.24 ± 3.60% (Figure 1D). In MCF-7 cells, treatment with 200 μg/mL citral decreased cell viability to 46.20 ± 6.04%, whereas treatment with 100 µg/mL and 200 µg/mL LEO, LEO-PEG, and LEO-Tw decreased cell viability to 46.58 ± 6.90% and 36.52 ± 7.08% (LEO), 39.33 ± 6.38% and 22.72 ± 4.04% (LEO-PEG), and to 40.25 ± 8.91% and 25.57 ± 4.72% (LEO-Tw) (Figure 1E).

**Figure 2:**
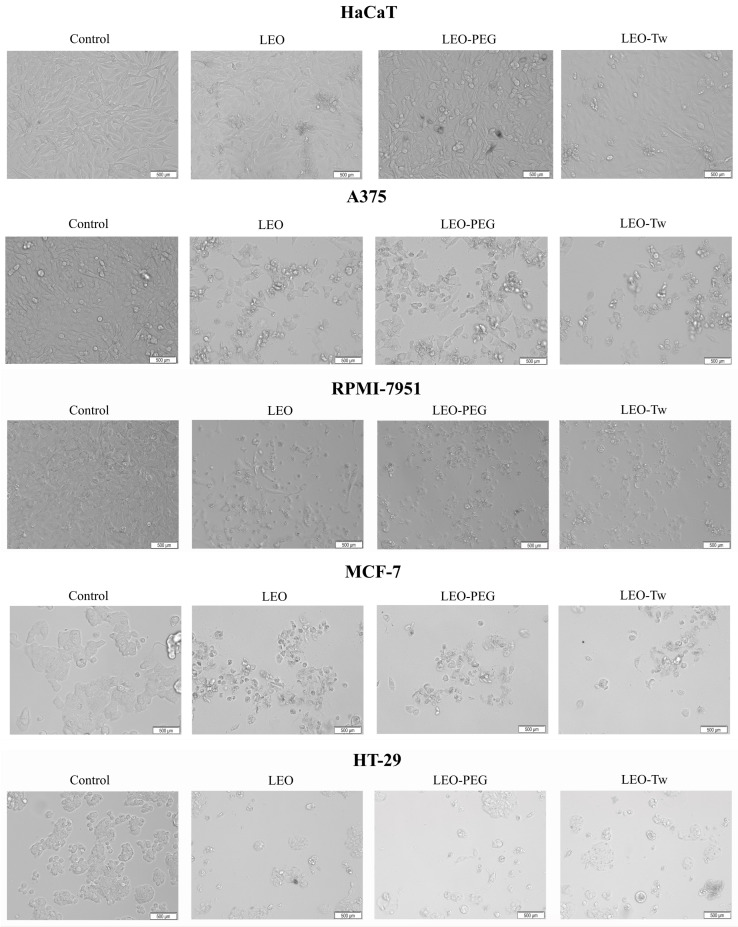
HaCaT, A375, RPMI-7951, MCF-7, and HT-29 cell line morphologies after 24 h treatment with LEO (1%), LEO-PEG, and LEO-Tw (200 μg/mL) vs. control.

The morphology of HaCaT cells remained unaltered following treatment with LEO, LEO-PEG, and LEO-Tw, showing preserved cell shape, adherence, and increased confluence, as observed in Figure 2. In contrast, all tested compounds induced marked cytotoxic effects in the cancer cell lines A375, RPMI-7951, MCF-7, and HT-29; the morphological assessment revealed characteristic signs of cell death, including loss of adherence, cell shrinkage, and rounding (Figure 2).

### 2.4. LEO Increases ROS Production

**Figure 3:**
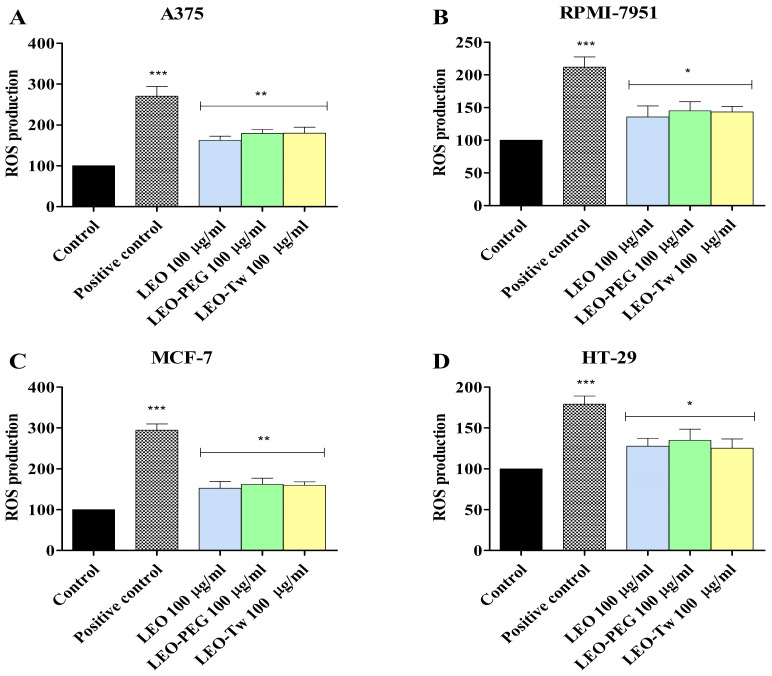
Assessment of ROS production in A375 (**A**), RPMI-7951 (**B**), MCF-7 (**C**), and HT-29 (**D**) cells exposed for 24 h to 100 μg/mL LEO, CEO-PEG, and CEO-Tw. *Tert*-butyl hydroperoxide (TBHP, 50 μM) was used as a positive control. The data represent the results of three independent experiments and are presented as the mean ± S.D (* *p* < 0.05, ** *p* < 0.01, and *** *p* < 0.001).

The ROS production in A375, RPMI-7951, MCF-7, and HT-29 cell lines after 24 h treatment with LEO, LEO-PEG, and LEO-Tw (100 μg/mL) was measured using the 2′,7′–dichlorofluorescein diacetate (DCFDA) assay. *Tert*-butyl hydroperoxide (TBHP, 50 μM) was used as a positive control. The results show that in A375 cell lines, treatment with LEO alone and formulated with PEG-400 and Tween 20 can significantly increase ROS production vs. control (100) as follows: 162.2 ± 10.11 (LEO), 178.7 ± 9.61 (LEO-PEG), and 179.8 ± 14.87 (LEO-Tw) (Figure 3A). A similar increase in ROS production vs. control was also observed after the treatment with LEO, LEO-PEG, and LEO-Tw in RPMI-7951 (135.4 ± 17.25, 145.1 ± 13.73, and 143.2 ± 8.58), MCF- 7 (152.5 ± 16.55, 162.0 ± 14.94, and 159.7 ± 8.35), and HT-29 (127.6 ± 9.60, 134.9 ± 13.14, and 125.1 ± 11.51) cell lines (Figure 3B–D).

### 2.5. LEO Decreases Mitochondrial Membrane Potential

**Figure 4:**
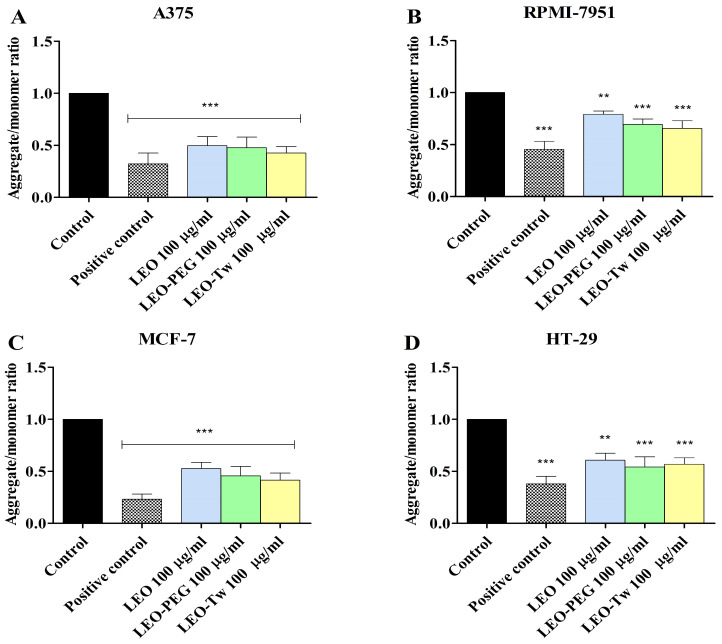
MMP, expressed as the JC-1 aggregate/monomer ratio of A375 (**A**), RPMI-7951 (**B**), MCF-7 (**C**), and HT-29 (**D**) cells exposed for 24 h to 100 μg/mL LEO, CEO-PEG, and CEO-Tw. FCCP (50 μM) was used as a positive control. The results are expressed as mean ± SD; n = 3 per group, ** *p* < 0.01, and *** *p* < 0.001.

In healthy mitochondria, the monomer form (green fluorescence) of the JC-1 dye enters the mitochondria and forms JC-1 aggregates that emit red fluorescence; in contrast, in dysfunctional mitochondria where the normal MMP is disrupted (depolarization), the JC-1 cannot enter the mitochondria, and the aggregate/monomer ratio (red/green form) decreases. Thus, the changes in the MMP were evaluated by measuring the aggregate/monomer ratio in A375, RPMI-7951, MCF-7, and HT-29 cells exposed for 24 h to 100 μg/mL LEO, CEO-PEG, and CEO-Tw (Figure 4). Treatment with LEO, LEO-PEG, and LEO-Tw decreased the aggregate/monomer ratio in all tested cell lines, thus suggesting that LEO essential oil can induce MMP depolarization. Specifically, the strongest effect was observed in the A375 cell line as follows: 0.49 ± 0.08 (LEO), 0.47 ± 0.10 (LEO-PEG), and 0.42 ± 0.06 (LEO-Tw) (Figure 4A). In comparison, in RPMI-7951, the effects were less pronounced, with LEO decreasing the aggregate/monomer ratio to 0.79 ± 0.03, LEO-PEG to 0.69 ± 0.05, and LEO-Tw to 0.65 ± 0.07 (Figure 4B). In HT-29, the aggregate/monomer ratio decreased to 0.60 ± 0.05 (LEO), 0.54 ± 0.09 (LEO-PEG), and 0.56 ± 0.07 (LEO-Tw), whereas in MCF-7, the values were 0.52 ± 0.05 (LEO), 0.45 ± 0.09 (LEO-PEG), and 0.41 ± 0.06 (LEO-Tw) vs. control (1) (Figure 4C,D).

### 2.6. LEO Effect on Mitochondrial Function

**Figure 5:**
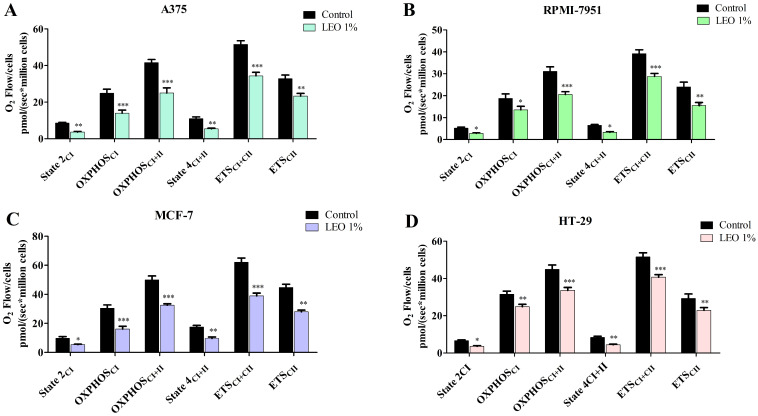
Mitochondrial respiratory rates of permeabilized A375 (**A**), RPMI-7951 (**B**), MCF-7 (**C**), and HT-29 (**D**) cells after treatment with 1% LEO. Results are expressed as mean values ± SD of three independent experiments (* *p* < 0.05, ** *p* < 0.01, and *** *p* < 0.001).

The mitochondrial respiratory rates of permeabilized A375, RPMI-7951, MCF-7, and HT-29 cancer cells after treatment with 1% LEO was assessed using high-resolution respirometry. In all tested cancer cell lines, LEO significantly decreased all oxygen consumption rates (Figure 5, Table 3).

### 2.7. Molecular Docking of LEO Components

In the current study, a molecular docking-based method was employed to determine possible cancer-related protein targets for the 17 LEO components. The theoretical inhibition of these protein targets by LEO components could be related to the LEO in vitro anticancer cytotoxic activity. Subsequently, we docked the aforementioned compounds into the binding site of druggable protein targets whose overexpression is often correlated with carcinogenesis, increased cell proliferation, and survivability within various types of cancer; the chosen protein targets for the in silico experiment were the vascular endothelial growth factor receptor 2 (VEGFR2), the epidermal growth factor receptor 1 (EGFR1), dual specificity mitogen-activated protein kinase 1 (MEK1), phosphoinositide-dependent kinase-1 (PDK1), phosphatidylinositol 4,5-bisphosphate 3-kinase catalytic subunit alpha isoform (PI3Kα), phosphatidylinositol 4,5-bisphosphate 3-kinase catalytic subunit gamma isoform (PI3K γ), mammalian target of rapamycin (mTOR), protein kinase B (AKT/PKB), apoptosis regulator Bcl-2 (Bcl-2), and apoptosis regulator Bcl-XL (Bcl-XL). The obtained docking scores for compounds 1–17 and the native ligands (NLs) for each target, used as positive controls, are given in Table 4.

**Figure 6:**
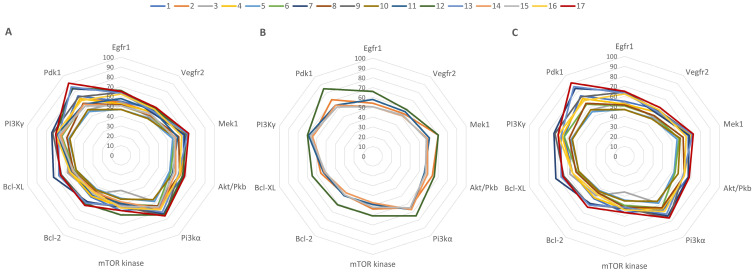
Radar graphs illustrating the docking scores of all docked components of LEO (**A**), its major constituents (**B**), and its minor components (**C**). The graph lines represent percentage values of each compound’s docking score relative to their respective NL docking score.

Docking scores were recorded as binding affinity values (kcal/mol), which implies that the lower the negative value, the greater the inhibitory potential of that compound. Neither of the docked compounds showed lesser binding affinities than the NLs, which served as positive controls. Nonetheless, to compare the 17 compounds’ combined effect against a specific protein target, all collected docking results were computed as a percentage of their respective NL’s docking result; these values were plotted as a radar graph, with each corner representing one of the 10 protein targets used. If an overall inhibitory tendency towards certain proteins is present, the graph should show lines (indicating affinity values) that are orientated closer to one or more corners (proteins) of the graph. In this situation, the compounds’ affinity lines are drawn closer to PDK1 and PI3Kα (Figure 6A). We divided the first graph into two subgraphs representing the major (compounds 2, 11, 12, 14, and 15) and minor components to see if the affinity trend holds in both situations. Figure 6B,C shows that there is a consistent preference for PDK1 and PI3Kα among LEO components. Having said that, among the major constituents, only compound 12 (β-caryophyllene) has a fairly substantial inhibitory potential for PDK1, scoring 85% of the native ligand’s docking score. However, the cumulative contribution towards PDK1 and PI3Kα is, to a higher degree, attributed to the effect of minor components such as compound 17 (β-caryophyllene oxide), 13 (humulene), or 7 (α-cubebene). β-caryophyllene oxide (BCO) was the most active theoretical inhibitor of both PDK1 and PI3Kα, excluding the NLs. This aspect is clearly visible from the graphs, where the line corresponding to compound 17 is depicted in dark red (Figure 6B,C). Moreover, apart from the case of PI3Kγ, Bcl-XL, and mTOR, the same structure was overall ranked as the highest theoretically active compound.

**Figure 7:**
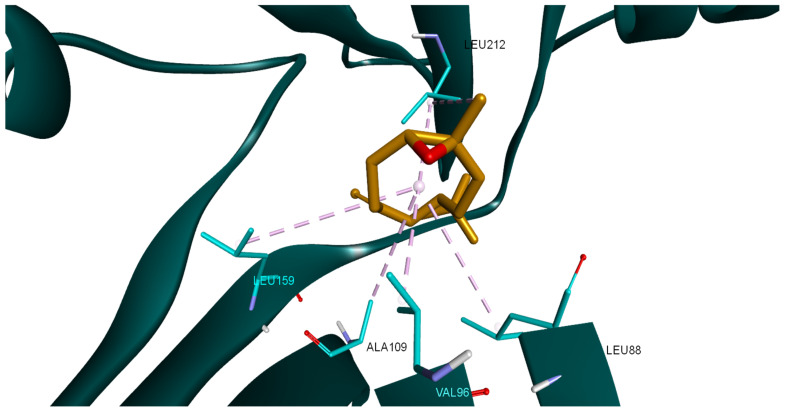
Compound 17 (β-caryophyllene oxide) docked into the binding site of PDK1 (PDB ID: 2PE1); hydrophobic interactions are depicted as purple dotted lines.

**Figure 8:**
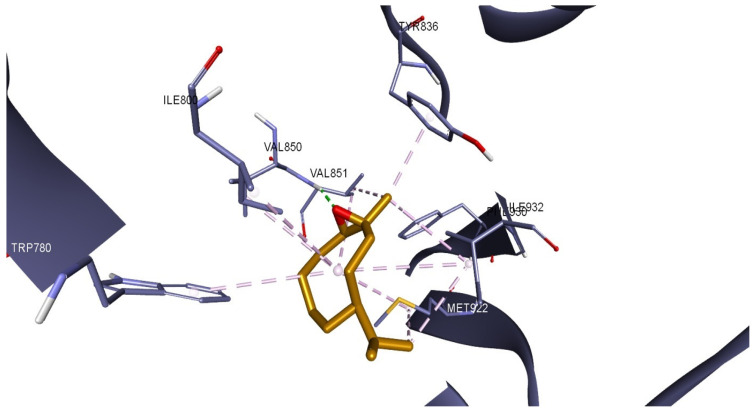
Compound 17 (β-caryophyllene oxide) docked into the binding site of PI3K α catalytic subunit (PDB ID: 6GVF); hydrophobic interactions are depicted as purple dotted lines, while hydrogen bonds are depicted as green dotted lines.

In the case of PDK1, β-caryophyllene oxide interacts with the binding site of the target protein exclusively through hydrophobic interactions with amino acids such as LEU88, VAL96, ALA109, LEU159, and LEU212 (Figure 7). The same compound exhibits a similar binding pattern when docked into the PI3Kα binding site. The molecule establishes multiple hydrophobic interactions with adjacent amino acids (TRP780, ILE800, TRP836, VAL850, VAL851, MET922, PHE930, ILE932), but it also interacts with the binding site via a hydrogen bond with VAL851. The interaction binding pattern is depicted in Figure 8.

## 3. Discussion

Cancer represents one of the main causes of mortality worldwide. Although 36 types of this pathology have been identified, the majority of cancer-related deaths are being caused by melanoma, breast, and colorectal cancer [12,13,14]. Melanoma represents the deadliest form of skin cancer due to metastasis formation and rapid development of drug resistance [12,15]. Similarly, the treatment of breast and colorectal cancer is often inefficient due to the appearance of chemoresistance and immunoresistance [16,17,18]. As a result, there is a search for new and advanced methods of treatment that would surpass the problem of drug resistance. In this context, medicinal plants have come forth as a new promising alternative [19]. In recent years, there have been several in vitro and in silico studies of plants and their products regarding anticancer effects [20]. Lemongrass emerged as a potential source of antitumor compounds due to its essential oil content, with 1–10% in plant floral tops and leaves, thus making lemongrass superior to other plants in terms of essential oil quantity [21,22]. The extraction method was chosen to suitably meet our purpose and infrastructure needs. The composition of the obtained essential oil was highly similar to other ones reported in terms of citral composition; other less occurring oil components may vary depending on several variables [21,23,24]. Even if other extraction methods such as microwave-assisted hydrodistillation (MAHD) or supercritical fluid extraction (SED) produce higher oil yields, the citral content does improve significantly and is dependent on other extraction factors [25]. The same study suggests that our chosen extraction method is the least expensive and environmentally harmful method for extracting lemongrass oil on a large scale. MAHD is appropriate for smaller operations, since it offers energy economy and shorter extraction times. Although SFE produces high-quality oil, it is more expensive and only works well in specific applications [25].

On the same note, DPPH scavenging activity of the LEO was not superior to the ones recorded for ascorbic acid or BHA but is in the same range as other values reported and can be mostly attributed to the high citral concentration [23,24]. LEO showed very close ABTS radical inhibition to both controls at a concentration of 1 mg/mL. This implies that LEO might be more efficient in scavenging ABTS radicals than DPPH radicals, possibly due to their difference in solubility and reactivity. A previous study showed that the major component, cital, showed higher free radical scavenging ability in the ABTS•+ assay than in the DPPH• assay [26]. Thus, citral’s antioxidant activity is more effectively determined by the ABTS method and is related to the same behavior exhibited by the LEO in the same scenario. The use of LEO could bring benefits to cancer therapies because of its biocompatibility, natural origin, and low cost [21]. Nevertheless, one of the disadvantages that essential oils have is their low solubility in water [22]. Therefore, in order to increase the homogeneity of essential oil–culture media mixture prepared in this study, we used Tween 20 and polyethylene glycol (PEG) 400. Tween 20 is a non-ionic surfactant with a HLB value of 16.7, thus forming emulsions with a suitable viscosity for drug formulations [27]. Also, there are multiple cytotoxic studies where Tween 20 was used for essential oil emulsification [27,28]. Likewise, PEGs are biocompatible non-toxic polymers often used as cosurfactants in pharmaceutical applications, PEG-400 being the most used subtype in self-emulsifying drug delivery systems [29,30,31,32]. After cell stimulation, it was observed that Tween and PEG-400 do not have any toxic effects on healthy and malignant cells, confirming their suitability for cytotoxic assays.

One of the main problems regarding cancer therapy is the poor selectivity for cancerous cells, with many side effects being caused by the toxic effects of the drug on healthy cells. Therefore, researchers aim to uncover highly selective molecules with anticancer properties [33]. In our case, neither one of the tested concentrations of LEO, LEO-PG, or LEO-TW significantly affected the viability of healthy keratinocytes, thus showing promising selectivity characteristics. Similar results were obtained by Al-Ghanayem et al., who reported that the cell viability of HaCaT cells was above 90% after treatment with *Cymbopogon flexuosus* essential oil (160 µg/mL, in culture media), the IC_50_ value being 1250 µg/mL [34]. On the contrary, the concentrations of LEO (0.5% and 1% *v*/*v*) that did not affect HaCaT cells showed toxic effects when tested on human dermal fibroblasts, the activity being dose-dependent [35]. This could be explained by the fact that different cell lines present different susceptibilities to tested compounds; also, the total concentration of citral (>89%) in the essential oil tested on fibroblasts was higher compared to our study (73.5%) [8,35].

On the other hand, all tested concentrations of LEO-CM, LEO-PEG, and LEO-TW presented cytotoxic effects on the colorectal adenocarcinoma, breast cancer, and melanoma cell lines. It was observed that higher concentrations (1% and 200 µg/mL) exhibited a stronger toxic effect compared to lower concentrations (0.5% and 100 µg/mL), with the anticancer activity being dose-dependent. A similar study was conducted by Sharma et al., who tested the effects of a LEO that was different in terms of chemical composition (*Cymbopogon flexuosus*) on HT-29 cells and other colon cancer cells (HCT-15, SW-620, and 502713). In agreement with our results, the study showed that lemongrass essential oil has a toxic effect on this type of cancer, with IC_50_ values of 42.4 µg/mL, 60.2 µg/mL, 28.1 µg/mL, and 4.2 µg/mL, respectively [7]. Whilst both oils shared some common components (linalool, citronella, and caryophyllene oxide), the major constituents in Sharma’s case were isointermedeol (24.97%), geraniol (20.08%), and geranyl acetate (12.20%) [36], while in our case, α-Citral (37.44%), β-Citral (36.06%), and linalool acetate (9.82%) were identified; despite their different compositions, both essential oils showed cytotoxic activity supporting the notion that multiple constituents have effects on cancerous cells [21,25]. Similar results were also described by Wang et al. [37], who revealed that Lemon myrtle (*Backhousia citriodora* F.Muell.) essential oil, rich in citral, (accounted for 74.9% of the content) had an IC_50_ value of 68.91 ± 4.62 on the HT-29 cell line. In a more recent study, the cytotoxic effect of LEO on HT-29 cells was attributed to citral and geraniol, compounds that were able to induce intrinsic apoptosis in malignant cells while leaving healthy cells unharmed [38]. α-citral, also known as geranial, and β-citral, known as neral, are aldehyde monoterpenes that form a racemic mixture (citral) which is considered an indicator of essential oil quality [21]. In recent years, there have been many studies that have focused on citral`s anticancer activity [39]. For example, Sheikh et al. revealed that citral has a dose-dependent toxicity on HT-29 (IC_50_ was 181.21 μM) and HCT116 (IC_50_ was 145.32 μM) after 24 h stimulation; moreover, the authors reported that cell death was induced through increased ROS levels and p53 activation caused by citral [13].

On melanoma cells, another study showed that citral has a cytotoxic effect, with IC_50_ values of 11.7 µM (SK-MEL-147) and 13.4 µM (UACC-257). The UACC-257 cell line, as well as the A375 line used in our study, harbors the BRAF mutation; the authors indicated that citral could reduce the effects of this mutation by interfering with the MAPK pathway and therefore interfere with carcinogenesis [15]. Aside from citral, other major components found in the LEO were researched for their anticancer potential. Several studies reported that linalool presents cytotoxic effects, the tested cell lines being those of colon cancer (WiDr, HCT-15, SW480 and RKO), lung cancer (A549), and ovarian cancer (HeyA8, A2780, and SKOV3ip1) [40]. While many studies have reported citral’s and linalool’s cytotoxic effects, it was often observed that essential oils have stronger activity compared to their individual constituents, possibly due to the synergistic relationship between the associated compounds [21]. These reports consolidate our findings, where the concentration of 200 μg/mL citral was less effective compared to the same concentration of LEO in all investigated scenarios. Research that confirms this observation was made by Gaonkar et al., who studied the effects of both *Cymbopogon flexuosus* essential oil and its components, citral and geraniol, in MCF-7 and HEK-293 cells. The authors also reported that the cell lines presented variant levels of sensitivity to tested compounds, with the MCF-7 cell line being more sensitive [8]. It is, therefore, safe to assume that anticancer activity is exerted through several molecular mechanisms shared by two or more individual components, with the association of complementary pathways being able to converge towards a synergistically improved overall anticancer outcome.

We investigated the underlying mechanisms of the LEO’s biological effects and identified its stimulatory activity on ROS production in all tested cancer cells. The induction of ROS by lemongrass oil was previously hypothesized by Lee et al., who assessed its antifungal activity [41]; in fact, its main component, citral, exhibited a similar ROS generation effect which was indicated as responsible for the significant anti-melanoma effects [15]. However, the intimate mechanism is more complex—on one hand, all stages in melanoma development involve oxidative stress and on the other, citral exerts cytotoxic effects through the induction of oxidative stress, as revealed by ROS generation. It is therefore safe to assume that the cytotoxic activity implies not only the generation of ROS but also a modulation of the intracellular pathways related to DNA damage, cellular proliferation, and death. In MCF-7 cells, our study revealed a stronger inhibitory effect on cell viability compared to previous data [42]; however, a more recent study reported significant anticancer activity against MCF-7 cells through mitochondrial depolarization and ROS generation [43]. Our results are in agreement with such studies, since we found a strong mitochondrial membrane depolarization in the A375, MCF-7, and HT-29 cells and at a lower degree in RPMI-7951, which is indicated as an important step of apoptosis. Changes in the mitochondrial morphology as a result of ROS production were previously noted in a different cell line of colon cancer, SW1417, where LEO induced mitochondrial fission in a dose-dependent manner, finally leading to apoptosis [44].

The increase in ROS production after LEO treatment can be further understood by evaluating the mitochondrial function, as the mitochondria are the primary site of ROS generation, specifically at the level of the complex I (CI) and complex III (CIII) of the electron transport chain (ETC) [45]. Increased ROS production in cancer cells has been associated with mitochondrial dysfunction, and the disruption of the mitochondrial ETC has been shown to lead to an increased ROS production in various cancer cell lines [46,47,48,49]. Our findings are similar; treatment with LEO increased ROS production and, at the same time, decreased all mitochondrial respiratory rates, suggesting that LEO can induce the impairment of mitochondrial function/mitochondrial dysfunction. Indeed, other studies have demonstrated that citral, the major component of LEO, has the ability to induce mitochondrial dysfunction by disrupting the tricarboxylic acid cycle (TCA) pathway and by damaging the mitochondrial membrane permeability of *Penicillium digitatum* cells [50]. Another study revealed that on the same P. digitatum cells, citral drastically affects all the mitochondrial complexes involved in oxidative phosphorylation, namely CI-CV. Moreover, citral significantly decreased intracellular ATP and the mitochondrial membrane MMP while increasing the accumulation of ROS [51]. In a similar manner, the current study showed that LEO induced mitochondrial dysfunction, increased ROS production beyond the threshold that cancer cells can tolerate, and decreased the MMP, ultimately leading to cell death. This dual nature, both promoting ROS production and being associated with antioxidant activity, is not unique to LEO and appears to be dependent on the cellular context and redox state. As revealed in the review by Bezzera et al. and in one of our recent studies, eugenol, the main component of clove essential oil, has similar antioxidant and pro-oxidant activities, acting as antioxidant in normal cells while displaying pro-oxidant cytotoxic effects in cancer cells depending on the cellular redox environment [52,53]. As previously mentioned, our experimental findings indicate that LEO exhibited superior cytotoxicity against cancer cells compared to citral. We also employed an in silico method to examine if the anticancer impact of LEO might be partially ascribed to the hypothesized suppression of active target proteins that promote carcinogenesis when overexpressed. Our docking results showed that among all the targets tested, PDK1 and PI3Kα were the preferred targets by the majority of LEO components. Minor components, rather than both citral isomers, were primarily responsible for the inhibition of these proteins. The strongest inhibitory potential for both proteins was attributed to CPO. Sadly, there are no experimental data regarding the biological inhibition of PDK1 by CPO. However, there is evidence that this compound targets PI3K. The results of Park et al. showed that CPO causes apoptosis by increasing mitochondrial ROS production in addition to inhibiting the constitutive activation of the PI3K/AKT/mTOR/S6K1 signaling pathway in PC-3 human prostate and MCF-7 breast cancer cells [54]. The inhibition activity of CPO may be closely related to its showcased binding pattern, which is similar to that of other PI3Kα inhibitors. Sapanisertib and a few other PI3Kα inhibitors, described in the study by Ouvry et al., form an essential hydrogen bond with the hinge region amino acid VAL851 [55]. The same interaction is present in the CPO-PI3Kα docked complex, which contributes to the compound’s high inhibition score among all other LEO constituents.

Thus, all presented findings indicate that LEO’s cytotoxicity could be attributed to the combined activity of its components and multiple modes of action. Effects on melanoma and colorectal cancer cells, along with good selectivity and possible reduction in drug resistance, all show that lemongrass essential oil has a potential as an anticancerous agent.

## 4. Materials and Methods

### 4.1. LEO Extraction and GC-MS Analysis

The dried plant material was received as a gift from “King Michael I” University of Life Sciences (Timisoara Romania Herbarium, voucher number VSNH.BUASTM-128). The dried plant was grounded and then was subjected to steam hydrodistillation for 4 h at 100 °C by means of a Craveiro-type apparatus [56,57]. As previously described [58], the steam was generated by heating a 3000 mL glass boiler equipped with electrical resistance and previously filled with water. The boiler was refilled whenever necessary. The steam was then transferred to the bottom of the glass extraction vessel (1000 mL). A water-cooling system was used to condense the steam and vaporized oil after it had passed through the plant material, which was placed on a perforated plate a few centimeters from the base of the extraction tank. Lastly, to prevent the creation of artifacts from overheating, LEO and hydrosol (aqueous phase) were collected in a 250 mL glass receiver fitted with a water-cooling jacket and a hydrosol overflow outlet. Following separation, the oil was treated with anhydrous sodium sulfate in order to remove water traces and stored at −18 °C in sealed vials for future analysis. The extraction yield was calculated according to the following formula: oil weight/dried plant weight × 100 (wt).

The sample was analyzed by gas chromatography using a GC Hewlett Packard HP 6890 Series gas chromatograph coupled with a Hewlett Packard 5973 Mass Selective Detector. Briefly, 1 μL of diluted sample (1:100 in hexane) was injected into the gas chromatograph under the following parameters: DB-WAX capillary column (30 m length, 0.25 mm internal diameter, 0.25 μm film thickness), a 50 °C to 250 °C temperature range with a rate of 6 °C/minute, and 4 min solvent delay. The mass spectrometer was set to 230 °C with the MS Quad at 150 °C and helium gas flow at 1 mL/min. The analyzed compounds ranged in mass from 50 to 600 amu. The resulting spectra were assessed against data from NIST 02 library (USA National Institute of Science and Technology software), and area percentage was determined. The retention indexes were calculated based on the retention times and areas of C9 to C18 alkanes; also, the Adams Indexes were used for comparation with literature.

### 4.2. DPPH Antioxidant Scavenging Activity Determination

The antioxidant activity of the samples was evaluated using the 2,2-diphenyl-1-picrylhydrazyl (DPPH) radical scavenging assay, as described by Rădulescu et al. [59]. A DPPH stock solution was prepared by dissolving 5 mg of DPPH in 5 mL of ethanol. Serial dilutions were then made in ethanol to generate a calibration curve with concentrations ranging from 7.81 µg/mL to 0.5 mg/mL. For comparative analysis, positive controls, ascorbic acid (AA), and butylated hydroxyanisole (BHA) were prepared in a concentration range between 0.06 µg/mL and 1.2 mg/mL.

The plant extract was diluted 1:10 in ethanol and mixed with a 0.25 mM DPPH ethanolic solution in a 1:4 (*v*/*v*) ratio. The reaction mixture was incubated in the dark at 25 °C for 30 min. Absorbance was measured at 515 nm using a Tecan Infinite 200Pro spectrophotometer (Tecan Group Ltd., Männedorf, Switzerland) with i-control software (version 1.10.4.0).

The percentage of DPPH radical inhibition (Inh%) was calculated using the following formula:

$$Inh\%=[(A_{0}-A_{s})/A_{0}]\times 100$$

where A_0_ is the absorbance of the control (DPPH solution without the sample) and A_s_ is the absorbance of the sample. The inhibition values were plotted against sample concentrations to determine the IC_50_ (half-maximal inhibitory concentration) using the calibration curve equation specific to each sample and control. The IC_50_ values, expressed in µg/mL, provide a quantitative measure of the sample’s antioxidant capacity, with lower values indicating higher antioxidant activity.

### 4.3. ABTS Radical Scavenging Assay

The ABTS [2,2′-azino-bis(3-ethylbenzthiazoline-6-sulfonic acid)] radical scavenging activity was assessed using a modified method [58,60]. To generate the ABTS cation (ABTS+•), a solution of ABTS (7.29 mM) was mixed in a 1:1 (*v*/*v*) ratio with K_2_S_2_O_8_ (2.47 mM) in an amber-colored bottle and kept in the dark at 25 °C for 14 h. The resulting ABTS+• solution was then diluted with ethanol to achieve an absorbance of 0.745 ± 0.047 at 734 nm. Next, 400 µL of the ABTS+• solution was combined with 100 µL of 1:10 diluted sample extract and incubated at room temperature in the dark for 30 min. The absorbance was subsequently measured at 734 nm using a 1 mg/mL solution of ascorbic acid (AA) and butylated hydroxyanisole (BHA) as reference compounds. The following equation was used:

%ABTS+• inhibition = (A_control_ − A_sample_) × 100/A_control_,

where A_control_ measures the absorbance of the ABTS+• solution mixture without adding the sample and A_sample_ measures the absorbance of the sample with ABTS+• solution mixture.

### 4.4. Cell Culture

Cell lines used for this study were immortalized human keratinocytes HaCaT, obtained from CLS Cell Lines Service GmbH (Eppelheim, Germany), and human melanoma cells A375 and human colorectal adenocarcinoma cells HT-29, both obtained from American Type Culture Collection (ATCC, Lomianki, Poland). HaCat and A375 cell lines were cultured in DMEM (Dulbecco’s Modified Eagle Medium) High Glucose, with the addition of 10% FBS (fetal bovine serum) and a 1% penicillin/streptomycin mixture (100 IU/mL), whereas HT-29 was cultured in McCoy’s 5A Medium, with 10% FBS and a 1% penicillin/streptomycin mixture (100 IU/mL). Cells were incubated in 5% CO_2_ atmosphere at 37 °C.

### 4.5. Cell Viability Assessment

Cell viability was determined using the Alamar Blue assay. Cell lines were seeded in 96-well culture plates (10^4^ cells/plate) and kept in an incubator at 37 °C and 5% CO_2_. After reaching 80–90% confluence, the old media was removed and the cells were stimulated using three methods as follows: (1) water dispersion method: lemongrass essential oil was mixed with culture media (LEO) to obtain 100 and 200 µg/mL that were used for cell treatment; (2) PEG method: lemongrass essential oil was mixed with PEG-400 (LEO-PEG) to obtain a 10 mg/mL mixture that was sonicated and thereafter diluted with culture media to obtain a 1 mg/mL concentration, and the respective mixture was diluted once more in culture media to obtain the final concentrations of 100 µg/mL and 200 µg/mL used for cell treatment; and (3) the Tween method, where Tween 20 (LEO-Tw) was used instead of PEG-400, with the remainder of the method being identical to the others. After 48 h of incubation with the test compound, the old medium was removed from the wells and the new medium containing Alamar Blue was added (final concentration 1.5%). Cells were incubated in standard conditions for another 4 h. Subsequently, the absorbance was measured at 570 nm using the xMark™ Microplate Spectrophotometer, Bio-Rad. Citral analytical standard, the main component of LEO, was purchased from Merck (Merck KGaA, Darmstadt, Germany) and also tested in the same conditions mentioned above at a concentration of 200 µg/mL.

### 4.6. Assessment of Cellular ROS Production

ROS production was evaluated using the cell permeant 2′,7′-dichlorodihydrofluorescein diacetate (H2DCFDA) kit (ab113851, Abcam, Cambridge, UK). After 24 h of treatment with 100 μg/mL citral, LEO, LEO-PEG, and LEO-Tw, the ROS production in A375, RPMI-7951, MCF-7, and HT-29 cell lines was quantified at Ex/Em at 485/535 nm using the BioTek Synergy HTX multimode microplate reader (Agilent Technologies, Santa Clara, CA, USA), following the protocol described by the manufacturer [61].

### 4.7. JC-1 Assay

The mitochondrial membrane potential was assessed using the JC1 Kit (JC1- Mitochondrial Membrane Potential Assay Kit ab113850, Abcam, Cambridge, MA, USA), according to the manufacturer’s specifications [62]. The JC-1 cationic dye exhibits a potential-dependent accumulation in mitochondria, as observed by the shift in fluorescence emission from green to red. The stained A375, RPMI-7951, MCF-7, and HT-29 cells treated with 100 μg/mL citral, LEO, LEO-PEG, and LEO-Tw were analyzed (Ex/Em: 535/590 nm) using a multimode microplate reader (BioTek Synergy HTX multimode microplate reader, Agilent Technologies, Santa Clara, CA, USA).

### 4.8. High-Resolution Respirometry

High-resolution respirometry was used to assess the mitochondrial function at 37 °C by means of the Oroboros high-resolution respirometer (Oxygraph-2k Oroboros Instruments GmbH, Innsbruck, Austria). A modified substrate uncoupler–inhibitor–titration (SUIT) protocol previously described by Petruș et al. was used to determine the mitochondrial respiratory rates [63]. A375, RPMI-7951, HT-29, and MCF7 cells were cultured until reaching confluence, trypsinized, counted, and resuspended (1 × 10^6^/mL cells) in a special mitochondrial respiration medium (EGTA 0.5 mM, 3 mM KH2PO4, taurine 20 mM, K-lactobionate 60 mM, MgCl_2_ 10 mM, D-sucrose 110 mM, HEPES 20 mM, and BSA 1 g/L, pH 7.1). The cells were added to the device chambers after 15 min in order to allow for the stabilization of the oxygen flux. In the first step, digitonin (20–35 μg/l × 10^6^ cells) was added to permeabilize the cell membrane, followed by glutamate (10 mM) and malate (5 mM) as the complex I (CI) substrates that allowed for the measurement of endogenous ADP-dependent basal respiration, also known as State 2. In the second step, ADP (5 mM) was added in order to enable the measurement of the CI (OXPHOSCI)-dependent active respiration, followed by the addition of succinate (10 mM) as the complex II (CII) substrate, thus enabling the measurement of active respiration dependent on both CI and CII (OXPHOSCI + II). Complex V was then inhibited with oligomycin (1 μg/mL), which resulted in the measurement of CI- and CII-dependent leak respiration (State 4). P-(trifluoromethoxy) phenylhydrazone carbonyl cyanide—FCCP—was further titrated in steps (1 μM/step) in order to achieve and measure the maximal respiratory capacity of the electron transport system (ETSCI + II). A CI inhibitor, rotenone (0.5 μM,), was then added to obtain the ETS dependent solely on CI. In the final step, antimycin A (2.5 μM) was added as a CIII inhibitor for the purpose of inhibiting the mitochondrial respiration and allowing for the measurement of the residual oxygen consumption (ROX). The final values were recorded following ROX correction.

### 4.9. Molecular Docking

Molecular docking studies were conducted following a previously described workflow [52]. Briefly, the protein target structures were searched in the RCSB Protein Data Bank [64] and optimized as docking targets by using Autodock Tools v1.5.6 (The Scripps Research Institute, La Jolla, CA, USA) (Table 5). The SDF structure files corresponding to the essential oil components were retrieved from PubChem [65] and converted into 3D structures by means of PyRx’s Open Babel module [66]. PyRx v0.8 [66] virtual screening software (The Scripps Research Institute, La Jolla, CA, USA) was employed in order to achieve molecular docking by using Vina’s encoded scoring function [67]. The validation of the docking protocol was conducted by the re-docking of the native ligands into their original protein binding sites. The root mean square deviation (RMSD) between the predicted and experimental docking pose of the native ligand was calculated in order to use the determined values to identify cases where molecular docking should be performed (a RMSD value not exceeding 2 Å was used as a threshold). The docking grid box coordinates and size were selected to best fit the active binding site (Table 5). Docking scores were calculated as ΔG binding energy values (kcal/mol). Protein–ligand binding interactions were analyzed using Accelrys Discovery Studio Visualizer 4.1 (Dassault Systems BIOVIA, San Diego, CA, USA).

### 4.10. Statistical Analysis

In order to determine the statistical differences compared to the control, the cell viability, ROS, and LP assay results were analyzed statistically using a one-way ANOVA followed by the Dunnett post-test. The high-resolution respirometry study results were analyzed using a two-way ANOVA with Bonferroni’s multiple comparison post-test. All the differences were considered to be statistically significant if *p* < 0.05 (* *p* < 0.05, ** *p* < 0.01, and *** *p* < 0.001).

## 5. Conclusions

The current study highlights the significant in vitro anticancer potential of LEO, exhibiting its cytotoxic efficiency against multiple cancer cell lines while maintaining its selective effect on non-cancerous cells. Further investigations showed that the cytotoxic effect of LEO was mediated through increased ROS production, mitochondrial membrane depolarization, and impaired mitochondrial function. Moreover, molecular docking analysis revealed that minor components, namely β-caryophyllene oxide, may play a significant role in targeting key proteins such as PDK1 and PI3Kα. These findings support that LEO’s anticancer activity is a cumulative effect attributed to several components as opposed to the major component, citral. While LEO represents a promising candidate for further investigations as an alternative or complementary anticancer therapy, future work encompasses in vivo anticancer efficiency validation and formulation strategies to enhance its bioavailability and therapeutic efficacy.

## Figures and Tables

**Table 1:** Chemical composition of LEO determined through GC-MS.

No	Compound Name	RT	RI Calc	AI	Area % Calc
1	Terpinolene	5.61	975		0.05
2	d-Limonene	5.96	994	1024	7.05
3	Eucalyptol	6.19	1005		0.12
4	*p*-Cymene	7.28	1063	1020	0.23
5	6-Methyl-5-hepten-2-one	8.61	1133		1.20
6	Citronellal	11.45	1283	1148	2.06
7	α-Cubebene	11.76	1299	1387	0.18
8	1,2,5,5-Tetramethyl-1,3-cyclopentadiene	12.07	1316		0.11
9	Triluoroacetyl-lavandulol	12.22	1324		0.18
10	Ethenyl-cyclohexane	12.74	1351		0.27
11	Linalyl acetate	13.30	1381	1254	9.82
12	β-Caryophyllene	13.86	1410	1418	6.73
13	Humulene	15.27	1484		0.93
14	β-Citral	15.53	1498	1316	36.06
15	α-Citral	16.48	1548	1338	37.44
16	Neryl acetate	16.80	1565	1359	2.43
17	β-Caryophyllene oxide	20.83	1778		0.66

**Table 2:** Scavenging antioxidant activity of LEO determined by DPPH and ABTS scavenging assays.

Sample	IC_50_ (µg/mL)	ABTS (Inh%)
LEO	92.30 ± 18.00	78.20 ± 0.040
AA	12.67 ± 5.28	88.84 ± 0.002
BHA	6.36 ± 13.56	88.79 ± 0.002

**Table 3:** The mean mitochondrial respiratory rates [pmol/(sec × million cells)] after the treatment of RPMI-7951, HT-29, A431, and NCI-H460 cells with eugenol and LEO.

		State 2	OXPHOS_CI_	OXPHOS_CI + II_	State 4	ETS_CI + II_	ETS_CI_
A375	Control	8.58	24.9	41.58	10.97	51.53	32.84
	LEO	3.64 **	13.97 *	25.03 ***	5.443 **	34.36 ***	23.32 **
RPMI-7951	Control	5.21	18.75	31.12	6.505	39.18	24.04
	LEO	2.84 *	13.56 ***	20.57 ***	3.31 *	28.79 ***	15.54 **
MCF-7	Control	9.69	30.45	50.02	17.49	62.03	44.67
	LEO	5.32 *	16.07 ***	32.53 ***	9.66 **	38.88 ***	27.89 **
HT-29	Control	6.603	31.56	44.90	8.40	51.60	29.32
	LEO	3.57 *	24.88 **	33.62 ***	4.35 **	40.72 ***	22.91 **

(* *p* < 0.05, ** *p* < 0.01, and *** *p* < 0.001).

**Table 4:** Recorded docking scores (binding affinity) for the 17 LEO components.

Compound	Protein Targets									
	Egfr1	Vegfr2	Mek1	Akt	PI3Kα	mTOR	Bcl-2	Bcl-XL	PI3Kγ	PDK1
	Binding Affinity (kcal/mol)									
NL	−10.9	−12.1	−9.4	−9.4	−8.8	−11.2	−11.3	−10.8	−9.3	−8.7
1	−6	−7	−6.3	−6.3	−5.8	−6.3	−5.9	−5.9	−5.9	−6.5
2	−5.9	−6.5	−6.6	−6	−5.7	−6	−5.5	−6	−6	−6.2
3	−5.9	−5.9	−5.4	−5	−5.1	−4	−5.5	−6.2	−5.2	−5
4	−5.7	−6.6	−6.6	−5.9	−5.7	−5.9	−5.8	−5.9	−6.1	−6.2
5	−5.1	−5.7	−5.2	−4.9	−5.1	−4.9	−4.8	−5.2	−5.2	−4.8
6	−5.5	−6.1	−5.4	−5	−5.6	−5.5	−5.1	−5.4	−5.9	−5.6
7	−7.1	−6.5	−6.4	−6.3	−6.5	−5.9	−6.6	−7.8	−6.9	−7.3
8	−5.6	−6.1	−5.8	−5.9	−5.6	−5.7	−5.1	−5.5	−5.4	−5.7
9	−7	−6.9	−6.7	−5.8	−6.8	−5.8	−6.8	−6.9	−6.8	−6.4
10	−5.1	−5.6	−5.5	−5.3	−4.9	−5	−4.9	−5.2	−5.1	−5
11	−6.3	−6.8	−5.7	−5.2	−5.9	−5.5	−5.6	−5.7	−6.4	−5.6
12	−7.2	−7.1	−6.6	−6.2	−6.6	−6.8	−6.9	−7	−6.5	−7.4
13	−6.9	−6.7	−6.7	−5.8	−6.3	−5.7	−6.8	−7.2	−6.2	−7.5
14	−5.5	−6.2	−5.5	−5.5	−5.9	−5.3	−5.2	−5.6	−6	−5.6
15	−5.5	−6.3	−5.4	−5.3	−5.7	−5.8	−5.5	−5.5	−6.2	−5.4
16	−6.8	−7	−6.1	−5.9	−6	−6	−5.3	−5.9	−6.5	−6
17	−7.1	−7.3	−6.8	−6.4	−6.5	−6.3	−7.1	−7	−6.5	−7.9

**Table 5:** Docking parameters used for the molecular docking of LEO components.

Protein (PDB ID)	Grid Box Center Coordinates	Grid Box Size	References
VEGFR2 (4ASD)	x = −23.4872	x = 14.6340	[68]
	y = −1.3964	y = 19.2181	
	z = −11.0618	z = 15.7983	
EGFR1 (1XKK)	x = 19.4479	x = 18.8523	[69]
	y = 33.9295	y = 18.8523	
	z = 38.3514	z = 18.8523	
MEK1 (3DV3)	x = 38.8352	x = 13.0549	[70]
	y = −14.6371	y = 19.2181	
	z = 0.0462	z = 11.5471	
PDK1 (2PE1)	x = −6.2833	x = 11.6743	[71]
	y = 44.2844	y = 14.1528	
	z = 44.0516	z = 11.6743	
AKT/PKB (4GV1)	x = −19.9894	x = 14.2711	[72]
	y = 3.3152	y = 13.5948	
	z = 11.0426	z = 15.4926	
PI3Kα (6GVF)	x = −17.2065	x = 14.8956	[55]
	y = 147.5732	y = 14.8956	
	z = 29.1217	z = 14.8956	
PI3Kγ (4FA6)	x = 44.2130	x = 13.5948	[73]
	y = 13.1865	y = 13.5948	
	z = 29.7323	z = 13.5948	
mTOR (4JSX)	x = 50.4459	x = 14.2711	[74]
	y = −2.0684	y = 13.5948	
	z = −48.5963	z = 15.4926	
BCL-XL (2YXJ)	x = −9.7398	x = 17.9927	[75]
	y = −16.3876	y = 26.4869	
	z = 8.8381	z = 15.9145	
BCL-2 (4LVT)	x = 7.6196	x = 16.5597	[76]
	y = −3.0737	y = 26.5085	
	z = −10.3894	z = 20.6849	

## Data Availability

The original contributions presented in the study are included in the article; further inquiries can be directed to the corresponding author.
